# Paranoia and Data-Gathering Biases in Autism

**DOI:** 10.1007/s10803-024-06301-w

**Published:** 2024-02-29

**Authors:** Kristina Bennert, Mark Brosnan, Amy Canning, Ged Roberts, Ailsa Russell

**Affiliations:** 1 Centre for Applied Autism Research, Department of Psychology, University of Bath, Bath, BA2 7AY UK; 2 Fromeside Secure Services, Avon and Wiltshire Mental Health Partnership Trust, Bristol, UK; 3 Specialist Nurse, Bristol Autism Spectrum Service, Bristol, UK

**Keywords:** Autism, Paranoia, Reasoning and decision making

## Abstract

Previous research has identified contradictory patterns in autism upon probabilistic reasoning tasks, and high levels of self-report paranoia symptoms have also been reported. To explore this relationship, the present study assessed 64 non-autistic and 39 autistic adults on two variants of a probabilistic reasoning task which examined the amount of evidence required before making a decision and ‘jumping to conclusions’ (a neutral beads task and an emotionally-salient words variant). The autism group was found to require significantly more evidence before making a decision and to have significantly less jumping to conclusions than the non-autistic group. For those with relatively low levels of paranoia, the emotionally-salient variant impacted on the non-autistic group, but not the autism group.

Paranoia, defined as the presence of unfounded beliefs that others intend to harm the self, exists on a continuum in clinical and non-clinical populations. Paranoid thoughts are regularly experienced by 10–15% of the general population and range from mild threat beliefs and ideas of reference to persecutory delusions which in their most strongly held, distressing and preoccupying form present symptoms of acute psychosis (Freeman, 2007). Psychological models of paranoia (Bentall et al., 2001; Salvatore et al., 2012) and delusions (Garety & Freeman, 1999) propose a number of cognitive mechanisms that contribute to the development and maintenance of persecutory delusions in psychosis and schizophrenia. These include (1) attentional and attributional biases (Bentall & Kaney, 1989; Bentall et al., 1994; Chadwick et al., 2005; Lyon et al., 1994); (2) theory-of-mind (ToM) deficits, i.e. difficulty with inferring others mental states (Frith & Corcoran, 1996); and (3) reasoning biases in the form of reduced data-gathering (Garety et al., 1991).

Autism is characterised by difference in social communication and interaction combined with a pattern of restricted and repetitive behaviours, interests and activities (American Psychiatric Association, 2013). While few studies to date have investigated paranoia in autistic people, a systematic review of seven empirical studies (Spain et al., 2016) found evidence for higher levels of paranoia in autistic people than the general population (up to 35%, see Ribolsi et al., 2022, for review). Ribolsi et al. (2023) review the contribution of attributional style and ToM to paranoia in autism and find that the evidence is contradictory. There is insufficient evidence to ascertain whether these factors contribute to the development and maintenance of paranoia and persecutory delusions in autistic people, or whether distinct autism-specific vulnerability factors may result in a qualitatively different cognitive structure of paranoia in autism (Pinkham et al., 2012; Spain et al., 2016). For example, it has been proposed that in autism, ToM impairments (Baron-Cohen et al., 1985) combined with frequent negative experiences of social interaction, rejection, victimisation and bullying, known to be high for autistic children (Schroeder et al., 2014), may give rise to persecutory ideation (see Spain et al., 2016). Whilst reasoning biases in the form of reduced data-gathering have been consistently associated with paranoia (So et al., 2016), little research has explored this relationship in autism.

Reduced data-gathering has most commonly been investigated with a probabilistic reasoning task known as the beads task (Phillips & Edwards, 1966). In this experimental task, individuals are shown two jars containing 100 beads each, in two colours with reverse proportions (e.g. 60 black and 40 white beads in one jar and 40 black and 60 white beads in the other jar). Participants are told that beads will be drawn from just one of the jars at random, shown to them and then returned to the jar. Participants can ask to see as many beads as they like up to a maximum of twenty, before making a decision as to which jar the beads come from. The most commonly measured variable using the beads task paradigm is draws to decision (DtD). This variable has been dichotomised into presence of a ‘jumping to conclusions’ (JTC) bias for individuals who request less than three beads (Garety et al., 1991). Logical equivalents of the beads task using positive and negative personality traits to describe a positive or negative person have also been developed to represent a more emotionally salient, social variation of the task (Dudley et al., 1997; Young & Bentall, 1997).

The JTC bias for people with psychosis symptoms is now a well-established phenomenon (Dudley et al., 1997; Huq et al., 1988; Young & Bentall, 1997) and has been targeted in cognitive-behavioural interventions (Moritz et al., 2010). Two meta-analyses of research based on the beads task have confirmed that strength of delusional ideation is negatively associated with data-gathering in healthy controls and clinical populations (Ross et al., 2015) but also suggest that the JTC bias is not a trans-diagnostic phenomenon beyond psychosis (So et al., 2016). It is important to understand whether the JTC bias is involved in the development and maintenance of paranoia in autistic people to ensure that cognitive-behavioural interventions can target the appropriate mechanisms (Spain et al., 2015).

The beads task was first examined in an autistic sample by Brosnan et al. (2014) who used the 60:40 ratio of the beads task with a group of autistic adolescents and age-matched controls. The autistic group requested significantly more beads than the control group and were characterised as having a ‘circumspect reasoning bias’. Thus, the data-gathering bias in autism was opposite to the data gathering bias in psychosis (Brosnan et al., 2010; 2013; Crespi & Badcock, 2008; see also Abu-Akel et al., 2015; 2018; 2022). A circumspect reasoning bias is consistent with the dual process theory of autism, which proposes that reasoning in autism is characterised by a dominance of deliberative (‘Type 2’) processing over a rapid and intuitive (‘Type 1’) processing, which has been demonstrated on a range of tasks (Ashwin & Brosnan, 2019; Brosnan et al., 2016, 2017; 2022; 2023; Lewton et al., 2019). In the beads task, the autistic group drew more beads than the control group before making their decision, but the groups were comparable in the level of confidence in their decision (Brosnan et al., 2014). However, Brosnan et al. (2014) did not assess levels of paranoia. Jänsch and Hare (2014) examined links between paranoia, autism and reduced data-gathering and found that the autistic group requested *less* beads than the control group on the 60:40 ratio version of the beads task. In addition, a third of the autistic sample showed a JTC bias, while no individuals in the non-clinical group showing this bias. While Jänsch and Hare (2014) also found significantly higher paranoia scores in the autistic group, the negative correlation between paranoia scores and draws to decision did not reach significance when the groups were analysed separately, though correlation coefficients showed a medium effect size (*r*>-.3) for the relationship between paranoia and draws to decision in each group.

The two studies exploring a data gathering bias in autism using the beads task therefore have contradictory findings, which may be related to co-occurring levels of paranoia within the autistic sample (see also Larson et al., 2015). The present study therefore compared an autistic and a comparison group on the beads task in addition to the more emotionally salient personality words variant of the task and included a measure of paranoia. The emotionally salient variant of the task has been found to encourage JTC (Dudley et al., 1997; Young & Bentall, 1997) and Dual Process Theory of Autism would predict this effect would not be present in the autistic sample (who rely on Type 2 processing). Dual Process Theory of Autism would therefore predict that the autistic group will draw more beads/ words (and fewer JTC). In addition, in line with Jänsch and Hare (2014), we would predict that the autistic group will have higher levels of paranoia than the comparison group and that this would correlate with JTC.

## Methods

### Participants

Participants for both autistic and comparison groups were recruited to take part in an online study via adverts on social media, including Twitter, Facebook groups, websites targeted at people with a diagnosis of autism, mailing lists of the National Autism Society and posters with QR codes displayed in public places. The exclusion criteria were: (1) under 18 years of age; (2) completion time < 5 min or > one hour; (3) multiple entries by the same participant; (4) autistic participants who identified as autistic but did not have a formal diagnosis of autism (from a clinician); non-autistic participants who screened positive for autism on the AQ10 (see below). 152 people accessed the web site, of which 126 completed the online survey (83%). 39 reported a formal diagnosis of autism (from a clinical professional) and 14 reported an informal (e.g. self) diagnosis of autism. 73 reported not having a diagnosis of autism, of which 9 scored above cut off on an autism screen, leaving 64 non-autistic participants who self-reported having no neurodevelopmental conditions.

### Measures

Widely used self-assessments of autistic traits and paranoid thoughts were selected for the study as they have been found to be valid and reliable for autistic and non-autistic samples. Similarly, two versions of the ‘jumping to conclusions’ task as they have been previously used on autistic and non-autistic populations were selected for the study – the beads task and the emotionally salient words task (see below). Draft versions of the study materials were piloted with three adults, including an autistic individual, to gauge approximate completion time, ensure intelligibility of materials and adjust the presentation of tasks to the needs of autistic individuals. The study took around 15–20 minutes. A range of 5 to 60 minutes was therefore set as responding too quickly or too slowly can be indicative of not attending to the task appropriately (see Revilla & Ochoa, 2017).

#### Demographic Information

: In addition to identifying if they were autistic, all participants were asked to provide information on their age, gender and highest educational qualification (none, GCSE typically taken at age 16; A-levels typically taken at age 18; undergraduate degree typically taken at age 21+; and post graduate qualification).

#### Autism-Spectrum Quotient Screen (AQ-10)

: The AQ10 is a brief ten-item questionnaire which was developed to provide a ‘red flag’ for autism sufficiently brief to be used in primary care practice (Allison et al., 2012). It is currently recommended as a screening tool in the NICE guideline on Diagnosis and Management of Autism Spectrum Disorder in Adults (CG142, 2012) in the UK, where a score of 6 or more is used as a cut-off. The questionnaire is composed of the top two most discriminatory items from the 50 item AQ (Baron-Cohen et al., 2001) and as such is not designed to be internally consistent. The AQ-10 has been reported to have a sensitivity of 79.87% [CI 95%: 69.13–90.60%] and a specificity of 87.31% [CI 95%: 76.87–95.52%]. It has been judged to perform reasonably similar to the full AQ-50 (Booth et al., 2013). The AQ-10 was included to enable screening out of possible undiagnosed cases of autism from the comparison group.

#### Green Paranoid Thoughts Scale (GPTS)

: The GPTS is a 32-item self-report measure composed of two subscales containing 16 items each, measuring ideas of social reference and persecution, respectively. It was developed to provide a measure of paranoia as a continuous variable in clinical and non-clinical populations (Green et al., 2008). Respondents are asked to rate each symptom statement on a scale ranging from 1 (“not at all”) to 5 (“totally”). Total score ranges from 32 to 160. The GPTS has been shown to have good test-retest-reliability (intraclass correlation coefficient = 0.87) and excellent internal consistency (Cronbach’s α = 0.90–0.95) in clinical and non-clinical populations alike (Green et al., 2008). In line with the continuum model of paranoia, there is no suggested clinical cut-off, but mean scores are reported as 48.8 (SD 18.7) for the non-clinical group and as 101.9 (SD 29.8) for the clinical group. The GPTS has been used in previous research examining the performance of autistic individuals on the beads task (Jänsch & Hare, 2014).

#### Beads Task

: This study adapted the 60:40 version of the beads task for computerised presentation (see Brosnan et al., 2014). The point at which participants opted to make a decision was recorded as a continuous variable (draws to decision, DtD). Decisions made after seeing less than three beads/words were classed as ‘jumping to conclusions’ and recorded as a categorical variable (JTC bias). After participants had chosen a jar, they were asked to rate how confident they felt in their decision by dragging a slider onto a value between 0 and 100. This was recorded as a continuous variable ‘confidence’. Completion time for the tasks was also recorded.

#### Words Task

: An emotionally salient and logically equivalent computerised version of the beads task was created based on the instructions and materials employed in two previous studies (Dudley et al., 1997; Young & Bentall, 1997). The survey task presents participants with a series of positive and negative personality trait words (e.g. generous, annoying). They are informed that the words are drawn at random from one of two surveys, one survey describing Person A, who is mostly liked and has been described with positive trait words by 60 out of a 100 people who took part in the survey, and Person B, who is mostly disliked and has been described with negative trait words by 60 out of 100 people. Participants were shown a visual representation of the two surveys and the order of positive and negative words was equivalent to the order of different coloured beads. The words task generated the same variables as the classic beads task to enable within-group comparisons.

In both versions of the task, the beads /words previously taken from the jar/survey were displayed in order at the bottom of the screen to eliminate the need for participants to remember previous draws. All materials were presented online using the Qualtrics online platform to ensure consistency of administration. Order of tasks was counterbalanced using the inbuilt randomisation function.

#### Ethics

: Ethical approval for the study was obtained from the University of Bath Psychology Research Ethics Committee.

### Analysis

Draws to decision is not typically a normally distributed variable (skewed to a low number of draws) and was therefore initial between group and correlational analyses using non-parametric statistics (Chi square X^2^, Mann-Whitney U and Spearman’s rho r_s_, respectively). Analysis of variance is not very sensitive to deviations from normality assumptions and a false positive rate is not affected by violation of this assumption (Glass et al., 1972; Harwell et al., 1992; Lix et al., 1996). Consequently, a repeated measures MANCOVA was conducted with draws to decision (beads and words) as the two dependent measures. As autism diagnosis was a dichotomous variable, paranoia was dichotomised into high and low using a median split separately for each group (autism group median = 48, non-autistic group median = 39). The independent variables were a diagnosis of autism (yes/ no) and paranoia (high/ low). Covariates of gender, age and highest qualification obtained were also entered.

## Results

Results from 39 autistic and 64 non-autistic participants were included in the analysis. Table 1 shows participant characteristics and differences between groups. There were no significant gender differences between the groups, though the autism group was significantly younger and had a lower level of highest education obtained. The autism group was also significantly higher in autistic traits and paranoia, see Table 1. For both the task variants (beads and words) the autism group had a significantly greater number of draws before making a decision, as well as significantly fewer JTC bias responses for both task variants, see Table 1. Despite greater draws to decision, the autism group did not take significantly longer on the tasks (non-autistic mean 37–38 s each task, autism mean 40–41 s each task; Beads: U = 1,393.0, z = 0.986, ns; Words: U = 1,395.0, z = 0.999, ns). There were also no significant group differences in confidence for the decision made (non-autistic mean 62–66; autism mean 66–68; Beads: U = 1,158.0, z = 0.613, ns; Words: U = 1,237.0, z = 0.146, ns). For both groups the draw to decision variable correlated highly for both task variants (non-autistic group: r_s_=0.72, *p* < .001; autistic group: r_s_=0.81, *p* < .001). For the non-autistic group, autistic traits significantly and positively correlated with paranoia (r_s_=0.40, *p* = .001) and this relationship was negative and non-significant for the autistic group (r_s_ = − 0.17, ns).

**Table 1:** Means (standard deviations, sd), medians (Inter Quartile Ranges, IQR) and percentages for the non-autistic (TD) group and the autistic group

	TD group (N = 64)	Autistic group (N = 39)	Tests for significance
Gender	Male 18 (28.1%) Female 26 (71.9%)	Male 17 (43.6%) Female 19 (48.7%) Other 3 (7.7%)	X^2^(1) = 3.69, ns
Mean age (years)	38.0 (sd = 11.9)	31.9 (sd = 13.0)	U = 859, z = 2.65, *p* < .01
Highest educational qualification	None 1 (1.6%) GCSE 7 (11.8%) A-levels 11 (17.7%) Degree 20 (32.3%) Postgrad degree 22 (35.5%) Other 1 (1.6%)	None 1 (2.6%) GCSE 5 (13.2%) A-levels 16 (42.1%) Degree 9 (23.7%) Postgrad degree 7 (18.4%) Other 0 (0%)	U = 850, z = 2.31, *p* < .05
AQ10 mean score	4 (IQR 3–4)	8 (IQR 7–8)	U = 2428, z = 8.13, *p* < .001
Paranoia median score (GPTS)	39 (IQR = 20)	48 (IQR = 32)	U = 1,594.5, z = 2.36, *p* < .05
Draws to decision: Beads	Mean 6.6 (sd = 5.3) Median 7.5 (IQR = 9)	Mean 9.5 (sd = 5.4) Median 9 (IQR = 7)	U = 1,595.5, z = 2.39, *p* < .05
Draws to decision: Words	Mean 6.2 (sd = 5.4) Median 6 (IQR = 10)	Mean 9.7 (sd = 5.8) Median 9.5 (IQR = 8)	U = 1,659.0, z = 2.84, *p* < .01
JTC bias: Beads	No 41 (64.1%) Yes 23 (35.9%)	No 34 (87.2%) Yes 5 (12.8%)	X^2^(1) = 6.54, *p* < .05
JTC bias: Words	No 37 (57.8%) Yes 27 (42.2%)	No 34 (87.2%) Yes 5 (12.8%)	X^2^(1) = 9.76, *p* < .01

**Fig. 1:**
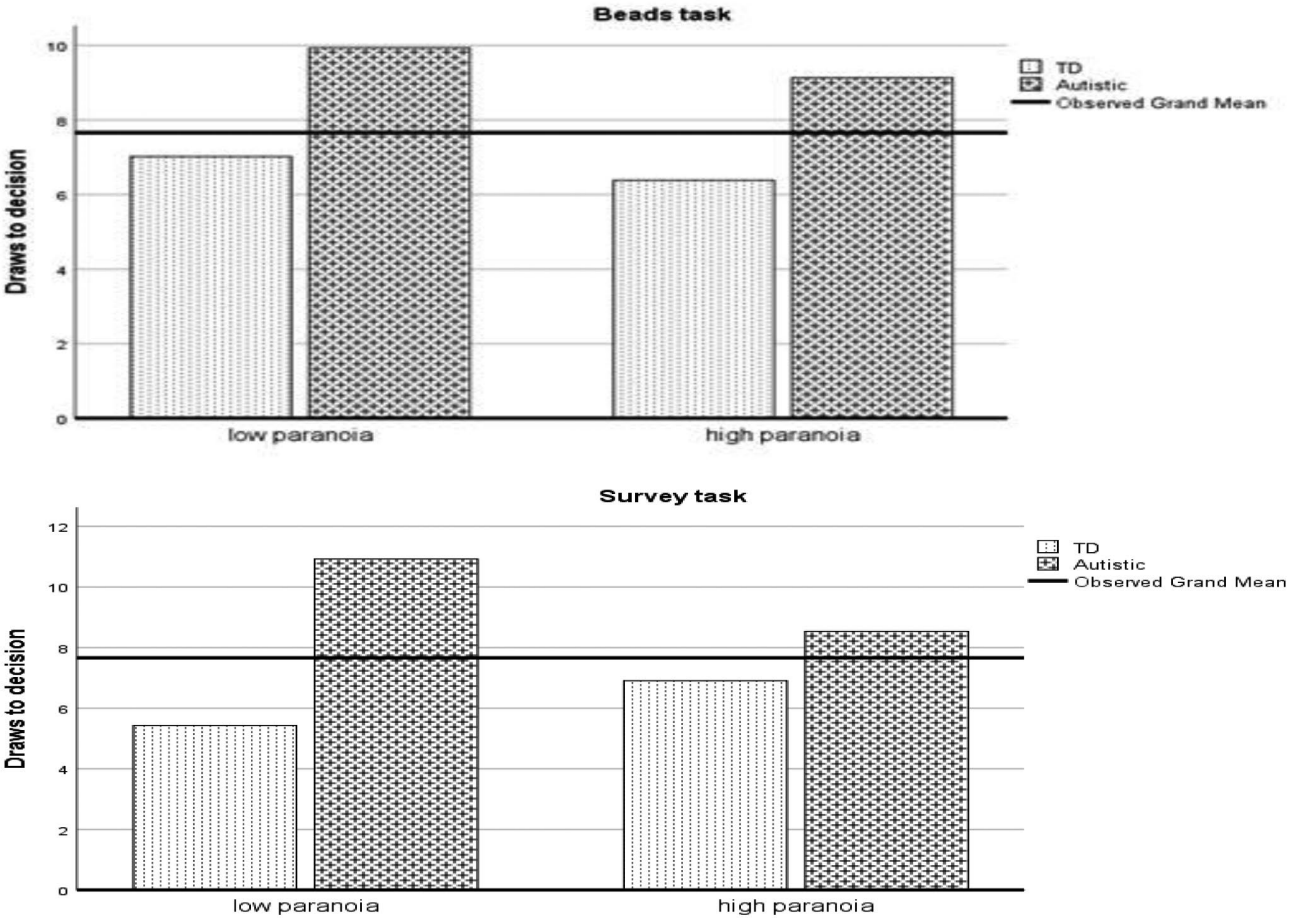
Draws to decision for non-autistic (TD) and autistic groups by high and low paranoia scores for survey-words and jar-beads tasks

MANCOVA was used to analyse the differences between the two variants of the task (Beads and Words) with reference to both autism diagnosis and paranoia (with covariates of gender, age and highest qualification, see analysis section above). Between groups, having an autism diagnosis was significant (F(1,93) = 8.57, *p* = .004) as was highest qualification (F(1,93) = 5.99, *p* = .016). Paranoia and the interaction between autism diagnosis and paranoia were both non-significant (*p* > .05) as were the covariates of gender and age (*p* > .05). A diagnosis of autism and a higher level of qualification (covariate) related to drawing more beads. Within groups, the task variant (beads/ words) was non-significant, as was its interaction with gender, age, highest qualification, autism diagnosis and paranoia (all *p* > .05). The 3-way task variant by autism diagnosis by paranoia interaction was significant, however (F(1,93) = 6.65, *p* = .012). The disparity between the autism group and the non-autistic group was greatest (autism group having more draws to decision) for the low paranoia group on the words variant of the task, see Fig. 1.

## Discussion

Paranoia and data-gathering style were investigated in 39 autistic adults and 64 non-autistic adults using two variants of a probabilistic reasoning task: the beads task and an emotionally salient equivalent (words task). Previous research has reported contradictory findings of autism groups drawing more beads before making a decision with a reduced JTC bias (Brosnan et al., 2014) as well as the opposite pattern – this difference possibly being explained by differing levels of paranoia (Jänsch & Hare, 2014). The present study found that, despite higher levels of paranoia, the autism group requested more information and were less likely to show a JTC bias than the non-autistic group on both variants of the task (beads and words). This is consistent with previous research concerning both paranoia (Spain et al., 2016) and the Dual Process Theory of Autism, which characterises reasoning within autistic groups as having a bias towards deliberative (Type 2) reasoning (Ashwin & Brosnan, 2019; Brosnan et al., 2014; 2016; 2017; 2022; 2023; Lewton et al., 2019). No differences in confidence about decisions were identified between the two groups, suggesting that autistic individuals make decisions at similar thresholds of certainty compared to non-autistic individuals, though requiring a greater amount of information to reach this level of certainty.

These findings are not consistent with Jänsch and Hare (2014) who report that their autistic sample displayed a marked JTC bias concluding that their findings ‘support the hypothesis that people with Asperger AS tend to make decisions on the basis of limited evidence’. The authors also report that their autistic group was likely to make counter-intuitive responses (e.g. choose the jar that did not match the colour ratio of the beads selected). This is inconsistent with accounts of decision making by autistic individuals which can be exhaustive as they strive to be logically consistent (De Martino et al., 2008; Luke et al., 2012). Overriding susceptibility to reasoning biases is also thought to require cognitive effort (Smith & Levin, 1996) and is demonstrated by autistic individuals (Fujino et al., 2019; Morsanyi et al., 2010; Shah et al., 2016), which is consistent with Dual Process Theory of Autism.

One potential explanation for the finding of a JTC bias in Jänsch and Hare’s (2014) autism sample, could be related to levels of paranoia, as paranoia has consistently been associated with JTC (Ross et al., 2015; So et al., 2016). In the present study, paranoia was not found to significantly impact upon draws to decision, whether analysed as a continuous variable (draws to decision) or a dichotomous variable (JTC). The autism group self-reported higher levels of paranoia as well as requesting more draws to decisions than the comparison group. These results are consistent with a data-gathering style not being a contributory factor for paranoia in autism, consistent with the proposal of a differential cognitive structure of paranoia in autistic individuals (Pinkham et al., 2012; Spain et al., 2016). This is also consistent with the suggestion that relationships between these variables demonstrated from clinical psychosis populations are not trans-diagnostic (So et al., 2016).

There was some evidence that autism diagnosis and levels of paranoia interacted with the task variants. The consistent finding of the autistic group drawing more beads before making a decision was most exaggerated in the words variant of the task for those with low levels of paranoia (see Fig. 1). The words task is proposed to be more emotionally salient, which results in more rapid decisions with fewer draws to decision (Dudley et al., 1997; Young & Bentall, 1997). This pattern was reflected in the non-autistic group but the opposite pattern was reflected in the autism group which is consistent with autistic participants not incorporating emotional context into their decision-making process resulting in greater logical consistency (De Martino et al., 2008) and the Dual Process Theory of Autism generally (Brosnan et al., 2016; 2017; 2022; 2023; Lewton et al., 2019). However, what is unclear is why this should be the case for the low paranoia group only. When comparing with the high paranoia group for emotionally salient version of the task, the autistic group made fewer draws to decision and the non-autistic group made more draws to decision. This relates back to the suggestion above that there may be a differential cognitive structure of paranoia in autistic individuals which warrants future research. Consistent with this, the present study also found a positive correlation between paranoia and autistic traits for the non-autistic group and a negative correlation between these variables for the autistic group. The present study is not consistent with Jänsch and Hare (2014) who found a reduced data-gathering bias in autism, except that this reduced data-gathering bias was identified for emotionally salient variations of the task for those with high levels of paranoia. It may be, therefore, that the inconsistency in previous research relates to the perceived emotional salience of the task or the cognitive structure of paranoia in autism.

This is pertinent as there are currently no appropriately validated instruments for measuring levels of paranoia in autism. While previous studies self-report measures have consistently reported higher levels of paranoia for autistic individuals, it may be that higher scores on these measures reflect autistic individuals beliefs and thoughts derived from actual experience of negative social interactions, bullying and marginalisation from peer groups (Maddox & White, 2015; Schroeder et al., 2014). Affirmative responses to questionnaire items that ask about experiences of being judged, being laughed at, and of others dropping hints, being hostile or trying to annoy the person (GPTS items A3, A4, A6, B5, B15) may potentially represent accurate descriptions of the social experiences of autistic individuals instead of indicating presence of ideas of reference and persecutory ideation. Freeman et al. (2008) also identify anomalous experiences as a possible predictor for the development of paranoia and the potential exists for autistic individuals to perceive sensory sensitivities as anomalous and respond accordingly. Future research needs to employ observational measures of paranoia (Freeman et al., 2008), and this extends to assessing autistic individuals.

There are a range of limitations to the present study. Firstly, the comparison group was different from the autistic group in size, age and level of academic achievement, although the findings of greater draws to decision were confirmed when accounting for these variables. Higher levels of academic achievement (covariate) also related to more draws to decision. This may be a proxy for general levels of intelligence, although a meta-analysis of 38 studies (Ross et al., 2015) examining the relationship between the JTC bias and delusions did not find evidence for an independent effect of IQ. Working memory deficits may affect task performance (Falcone et al., 2014) and working memory load was minimised by providing a record on the screen of previous draws. The sample was also unbalanced between autistic and non-autistic participants, and the relatively small sample size may also limit the generalisability of the findings. Overall, the sample was well-qualified which limits the generalisability of the findings, as no participants reported having an intellectual disability. In addition to the questionnaire measures being self-report (see above), the diagnostic status of participants could not be verified and should therefore be considered self-report rather than independently verified. Participants were given the opportunity to indicate if they had a formal diagnosis (from a clinician) or an informal (e.g. self) diagnosis. This latter group was excluded from the analysis in an attempt to ensure as far as possible that the autistic group comprised of those with a formal clinical diagnosis. In addition, those from the non-autistic group who scored above cut-off on the AQ10 were not included to exclude any undiagnosed autistic adults from this group as far as possible. The AQ10 is not diagnostic, although the group means for the non-autistic and autistic groups were below and above cut-off, respectively. Generally, the online nature of the study may have impacted upon participants responses, though the similarity to the draws to decision of previous research is note-worthy (non-autistic group = 6; autistic group = 10; Brosnan et al., 2014). Finally, autistic and non-autistic groups were targeted for recruitment, not groups with clinical levels of paranoia. Paranoia was dichotomised based upon group medians and this method as well as the range of paranoia scores needs to be borne in mind when interpreting the findings and future research can extend to groups with clinical levels of paranoia.

In conclusion, the findings are consistent with research that characterises autism as being associated with a data gathering bias, that is a propensity for greater data gathering before making a decision. Whilst higher levels of paranoia are associated with a reduced data gathering bias in the non-autistic population, this is not the case in the autistic population. This is consistent with the proposal of a differential cognitive structure of paranoia in autistic individuals which warrants future research. Future research can also identify the situations when a propensity for greater gathering can lead to *better* decision making, and potentially an autism-related strength.
